# INTERDISCIPLINARY DEMENTIA CARE WORKFORCE TRAINING DURING COVID-19 AND TWO HOSPICE CASE STUDIES

**DOI:** 10.1093/geroni/igac059.338

**Published:** 2022-12-20

**Authors:** Rebecca Lassell, Aditi Durga, Shih-Yin Lin, Tessa Jones, Ariel Ford, Abraham Brody

**Affiliations:** New York University, New York, New York, United States; New York University, New York, New York, United States; New York University, New York, New York, United States; New York University, New York, New York, United States; New York University Rory Meyers College of Nursing, New York, New York, United States; HIGN at the NYU Rory Meyers College of Nursing, New York, New York, United States

## Abstract

Hospice is a care model characterized by interdisciplinary team-based, person and family-centered care. To optimize agency-wide interdisciplinary team-based hospice dementia care, at least two levels of tailoring of the dementia care workforce training are imperative, first, by discipline, and second, by hospices’ local culture and needs. As of February 2022, a thousand and one skilled hospice interdisciplinary team members (not counting champions) across 18 hospice agencies have completed their discipline-specific Aliviado dementia care training, including 56 providers, 763 nurses, 129 social workers, and 53 chaplains. In this presentation, we describe how we tailored dementia workforce training for skilled interdisciplinary team members (first level tailoring), as well as provide two case studies elucidating how we performed further tailoring of the program for two large hospice agencies (average daily census: 354 and 868, respectively) in two different states to meet their local needs (second level tailoring), and lessons learned.

